# The role of the master cancer regulator Pin1 in the development and treatment of cancer

**DOI:** 10.3389/fcell.2024.1343938

**Published:** 2024-04-30

**Authors:** Robert Stewart, Shaunik Sharma, Timothy Wu, Sho Okuda, George Xie, Xiao Zhen Zhou, Brian Shilton, Kun Ping Lu

**Affiliations:** ^1^ Department of Biochemistry, Western University, London, ON, Canada; ^2^ Robarts Research Institute, Western University, London, ON, Canada; ^3^ Department of Pathology and Laboratory Medicine, Western University, London, ON, Canada; ^4^ Lawson Health Research Institute, Schulich School of Medicine and Dentistry, Western University, London, ON, Canada; ^5^ Department of Oncology, Western University, London, ON, Canada

**Keywords:** peptidyl–prolyl isomerase, Pin1 inhibition, cancer therapy, cancer stem cells, epithelial–mesenchymal transition, immunosuppression, oncogenesis, combination therapy

## Abstract

This review examines the complex role of Pin1 in the development and treatment of cancer. Pin1 is the only peptidyl–prolyl isomerase (PPIase) that can recognize and isomerize phosphorylated Ser/Thr-Pro peptide bonds. Pin1 catalyzes a structural change in phosphorylated Ser/Thr-Pro motifs that can modulate protein function and thereby impact cell cycle regulation and tumorigenesis. The molecular mechanisms by which Pin1 contributes to oncogenesis are reviewed, including Pin1 overexpression and its correlation with poor cancer prognosis, and the contribution of Pin1 to aggressive tumor phenotypes involved in therapeutic resistance is discussed, with an emphasis on cancer stem cells, the epithelial-to-mesenchymal transition (EMT), and immunosuppression. The therapeutic potential of Pin1 inhibition in cancer is discussed, along with the promise and the difficulties in identifying potent, drug-like, small-molecule Pin1 inhibitors. The available evidence supports the efficacy of targeting Pin1 as a novel cancer therapeutic by analyzing the role of Pin1 in a complex network of cancer-driving pathways and illustrating the potential of synergistic drug combinations with Pin1 inhibitors for treating aggressive and drug-resistant tumors.

## Introduction

Peptidyl–prolyl peptide bonds are special in that both the *cis* and *trans* conformations can exist in biologically relevant proportions. In non-prolyl-containing peptide bonds, steric hindrance in the *cis* conformation makes the *cis* isomer energetically unfavorable compared to the *trans* isomer, which represents nearly 99.9% of non-prolyl peptide bonds at equilibrium (Wedemeyer et al., 2002). With prolyl residues, the lack of intramolecular hydrogen bonding and the presence of the pyrrolidine ring create steric constraints in the *trans* isomer, raising the energy of the *trans* form to a level similar to that of the *cis* form so that both *cis* and *trans* isomers exist in significant proportions at equilibrium (Schmidpeter and Schmid, 2015; Dunyak and Gestwicki, 2016). Isomerization of peptidyl–prolyl bonds can result in whole-protein conformational changes and is considered the rate-limiting step in protein folding, occurring quite slowly (on a scale of seconds) when uncatalyzed (Wedemeyer et al., 2002). This explains the existence of a large family of peptidyl–prolyl isomerases (PPIases) that catalyze the *cis*/*trans* isomerization of peptidyl–prolyl bonds (Dunyak and Gestwicki, 2016). The PPIase superfamily includes cyclophilins, FK506-binding proteins, and parvulins, which generally act as protein-folding chaperones, regulating the activation and/or stability of their targets (Rein, 2020). PPIases have diverse roles throughout the cell, including cellular trafficking, T-cell activation, immunoregulation, apoptosis, cell growth, and differentiation (Wang and Heitman, 2005; Nigro et al., 2013; Tong and Jiang, 2015).

Unique among the PPIases is “peptidyl–prolyl isomerase never in mitosis gene A (NIMA) interacting-1," otherwise known as “Pin1.” Pin1 is the only known PPIase—among the ∼30 PPIases in the human proteome—that specifically recognizes and catalyzes the isomerization of phosphorylated Ser/Thr-Pro motifs (p-Ser/Thr-Pro) (Ping Lu et al., 1996; Lu and Zhou, 2007). Pin1 works in concert with protein kinases and phosphatases that phosphorylate and dephosphorylate Ser/Thr-Pro motifs, respectively. This cooperation controls the activity and/or stability of their protein targets (Lu and Zhou, 2007; Means and Yeh, 2007).

Pin1 is a small (18.2 kDa) protein, containing 163 amino acids that comprise an N-terminal WW domain (residues 1–39) and a C-terminal PPIase domain (residues 50–163) connected by a flexible linker (residues 40–49). The C-terminal PPIase domain is responsible for catalyzing the isomerization of Pin1 substrates. It folds into a four-stranded β-sheet surrounded by four α-helices that form a globular domain with a shallow depression for the active site (Ranganathan et al., 1997). A trio of conserved catalytic residues point outward and create a binding pocket for proline and the peptide bond of the Pin1 substrate, namely, Leu-122, Met-130, and Phe-134 (Ranganathan et al., 1997). The PPIase domain also contains a basic pocket created by Lys-63, Arg-68, and Arg-69 that directly binds the substrate phosphate (Xu et al., 2014). The most important catalytic residue is Cys-113, which is thought to have a very low pKa that imparts a negative charge at neutral pH. Pin1 with a mutation of Cys-113 to Asp retains catalytic activity and cellular function, consistent with the presence of Asp at an equivalent position in related members of the parvulin family (Behrsin et al., 2007). On this basis, binding of the p-Ser/Thr-Pro substrate to the Pin1 PPIase domain is thought to accelerate isomerization by decreasing the double-bond character of the peptide bond through destabilizing interactions with the negative charge on the Cys-113 thiolate in Pin1 (Behrsin et al., 2007). The WW domain consists of three anti-parallel β-strands that form a hydrophobic surface, including two conserved tryptophan residues, which bind to p-Ser/Thr-Pro motifs (Lu et al., 1999; Kato et al., 2002). The fact that the WW domain of Pin1 binds to the same p-Ser/Thr-Pro motifs that serve as substrates for the isomerization domain raises questions about the roles of the WW domain in the physiological functions of Pin1. A comprehensive discussion of the Pin1 function with an emphasis on the roles of the WW domain and the interplay between the WW and PPIase domains can be found in Lee and Liou (2018).

In summary, Pin1 is a flexible, sequence-specific, and phosphorylation-dependent enzyme that recognizes, binds, and isomerizes p-Ser/Thr-Pro motifs and that can impact the activity, stability, and subcellular location of interacting proteins, playing important roles in cell cycle regulation, differentiation, immune regulation, stemness, and tumorigenesis (Zhou and Lu, 2016).

## Cancer and the role of Pin1 in phosphoregulation

Cancer is an umbrella term for a large variety of complex diseases categorized by their tissue or cell of origin and individual molecular alterations (Brown et al., 2023). In the simplest terms, cancer can be defined as a fundamental inability to regulate cellular proliferation (Brown et al., 2023). However, tumorigenesis is a multi-step process that requires the acquisition of numerous ‘cancer hallmarks’—like immune evasion and replicative immortality—which allow for successful growth to occur (Hanahan, 2022). Functionally, Pin1 contributes to the acquisition of at least 10 of Hanahan and Weinberg’s cancer hallmarks: sustaining proliferative signaling, evading growth suppression, enabling replicative immortality, activating invasion and metastasis, inducing angiogenesis, avoiding immune destruction, deregulating cellular energetics, genomic instability, tumor-promoting inflammation, and evading apoptosis (Chen et al., 2018). Through stabilizing or destabilizing its target, Pin1 can increase the activation of over 70 oncogenes as well as inactivate over 30 tumor suppressors (Chen et al., 2018). The uncontrolled propagation of growth signals in cancer is mediated by the phosphorylation of 13,000 phosphoproteins with 230,000 phosphosites (Sacco et al., 2012; Li X. et al., 2013). Protein phosphorylation affects protein surface properties, conformation, and stability, thereby altering protein activity, protein–protein interactions, and/or subcellular location (Humphrey et al., 2015). Phosphorylation is essential in the regulation of almost all cellular processes, including protein synthesis, proliferation, metabolism, cell division, aging, and apoptosis (Lee et al., 2011a; Lake et al., 2016; Gerritsen and White, 2021). As the magnitude, duration, and location of phosphorylation are crucial for determining specific biological functions, phosphorylation is tightly regulated by many positive and/or negative feedback mechanisms (Lake et al., 2016). However, in the context of tumor cells, this meticulously orchestrated mechanism becomes dysregulated and directly promotes oncogenesis, often through the alteration of the kinase function (Gerritsen and White, 2021).

Two major classes of kinases—serine/threonine kinases and tyrosine kinases (TKs)—are responsible for facilitating all phosphorylation events throughout the cell. Both Ser/Thr and TKs have been implicated in various disease processes but are probably best known for their diverse roles in various cancers. Although TKs like HER2 and EGFR are instrumental for the transformation of specific malignancies like breast and lung cancers, most of the cell’s phosphorylation-dependent fate relies on Ser/Thr kinases (Lee et al., 2011a; Chen and Igumenova, 2023).

**FIGURE 1:**
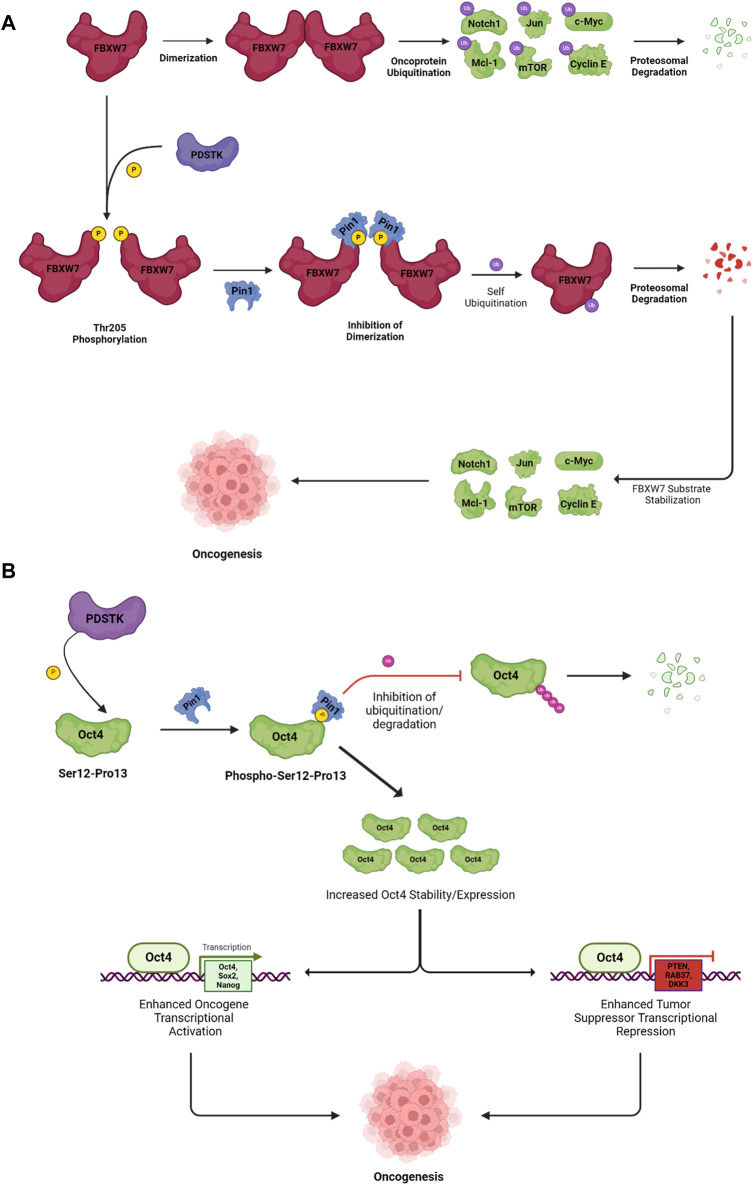
Pin1 promotes the proteasomal degradation of tumor suppressor protein FBXW7 and stabilizes oncogenes to promote oncogenesis. **(A)** PDSTK-mediated phosphorylation on the Thr205-Pro206 motif on tumor suppressor protein FBXW7 facilitates interactions with Pin1, which inhibits FBXW7 dimerization, resulting in self-ubiquitination and subsequent proteasomal degradation. Downstream FBXW7 substrates such as Notch1, Jun, and c-Myc are stabilized, which promote oncogenesis. **(B)** Pin1 interacts with the oncoprotein Oct4 following its PDSTK-mediated phosphorylation. Pin1 increases Oct4 stability by inhibiting ubiquitination and degradation, resulting in enhanced transcriptional activity. Increased Oct4 inhibits the transcription of tumor suppressors and promotes oncogene transcription; together these effects promote oncogenesis. Abbreviations: FBXW7, F-box and WD repeat domain containing 7; Notch1, neurogenic locus notch homolog protein 1; Jun; c-Myc, c-myelocytomatosis oncogene; Mcl-1, myeloid cell leukemia sequence 1; mTOR, mammalian target of rapamycin; Oct4, octamer-binding transcription factor 4; Sox2, SRY-box 2; PTEN, phosphatase and tensin homolog; RAB37, member Ras oncogene family 37; DKK3, dickkopf-3. The figure was generated using BioRender^®^.

A subset of Ser/Thr kinases, known as proline-directed serine/threonine kinases (PDSTKs), takes center stage in orchestrating oncogenesis by regulating cell-cycle progression in a wide variety of cancers (Lee et al., 2011a). As the name suggests, PDSTKs have specificity for Ser/Thr residues directly preceding a proline (Ser/Thr-Pro motifs) (Chen and Igumenova, 2023). Members of this class include mitogen-activated protein kinases (MAPKs), cyclin-dependent kinases (CDKs), stress-activated protein kinases/c-Jun N-terminal kinases (SAPKs/JNKs), p38 kinase, polo-like kinases (PLKs), glycogen synthase kinase 3 (GSK3), dual-specificity tyrosine-regulated kinases (DYRKs), homeodomain-interacting protein kinases (HIPKs), SR protein-specific kinases (SRPKs), and CDC2-like kinases (CLKs) (Wang et al., 1998; Boni et al., 2020; Steinmetz et al., 2021; Johnson et al., 2023; ScienceDirect Topics, 2023). Pin1 is the only prolyl isomerase able to catalyze phosphorylated Ser/Thr-Pro motifs; the *trans* and *cis* forms of the pSer/Thr-Pro motifs have different effects on enzyme activity, protein interactions, stability, cellular location, and dephosphorylation by regulatory phosphatases. An example of how phosphorylation on a Ser/Thr-Pro motif and interaction with Pin1 can have broad effects on protein stability is illustrated in Figure 1A. Here, the E3 ligase subunit, F-box and WD repeat domain containing 7 (FBXW7), is active as a dimer and ubiquitylates several oncoproteins, leading to their proteasomal degradation. When phosphorylated on Thr-205, which is followed in sequence by Pro-206, the Pin1 interaction leads to an inhibition of dimerization, followed by auto-ubiquitylation and degradation of FBXW7, increasing the stability of several oncoprotein targets (Min et al., 2012).

## Mechanisms of Pin1 dysregulation in cancer

### Pin1 overexpression

As Pin1 is the only phosphorylation-dependent prolyl isomerase, it is solely responsible for regulating the substrates of a vast array of cancer-promoting kinases. Accordingly, Pin1 overexpression is correlated with poor clinical outcomes in numerous cancers (Zhou et al., 2016). The overexpression of Pin1 can be attributed to both upstream oncoproteins and a positive feedback loop arising from Pin1 activity; Pin1 activates pathways that increase its expression and inactivates pathways that decrease its expression. Several tumor suppressors like p53, BRCA1, PKA, and DAPK1 negatively regulate Pin1 expression/activation (Chen et al., 2018). E2F and neurogenic locus notch homolog protein 1 (Notch1) transcription factors induce Pin1 expression and are known to play an important role in cancer progression by regulating S-phase entry and differentiation, respectively (Ryo et al., 2002; Rustighi et al., 2009). Thus, upstream activators of Pin1 expression involve oncoproteins responsible for regulating cell-cycle progression: HER2, Ras, cyclin D1, p38, and PI3K (Liu et al., 2016). Not only is Pin1 expression activated by several oncoproteins, but it also promotes the expression/activation of E2F and Notch1/4 transcription factors, thereby directly contributing to positive feedback on Pin1 expression (Rustighi et al., 2009; Lin et al., 2015). Moreover, Pin1 targets both c-Jun and c-Fos to increase AP-1 transcriptional activity, which directly promotes the expression of cyclin D1 (Lu et al., 2007). Pin1 also directly interacts with and stabilizes cyclin D1 and RB, thus promoting the activation of CDK4/6 and RB hyperphosphorylation; this, in turn, induces E2F expression and, subsequently, expression of Pin1 (Tong et al., 2015; Cheng and Tse, 2018). Pin1-mediated activation of CDK4/6 is also responsible for promoting Pin1 stability by phosphorylating and inactivating the anaphase-promoting complex (APC/C^cdh1^), an E3 ligase that, when unphosphorylated, targets Pin1 for degradation in the G1 phase and restrains S-phase entry. Following CDK4/6-mediated phosphorylation, APC/C^cdh1^ is inactivated by Pin1-mediated isomerization (Ke et al., 2024). Pin1 also negatively regulates FBXW7 (Figure 1A), an E3 ligase responsible for the degradation of numerous oncoproteins, including Notch1 (Lu et al., 2007). Decreased FBXW7 activity thus stabilizes Notch1, which further promotes Pin1 expression.

### Pin1 post-translational modifications

Pin1 can also be dysregulated in cancer through post-translational modifications like phosphorylation and SUMOylation. In the case of phosphorylation, oncogenic kinases drive Pin1 activation, while tumor suppressive kinases inhibit it. Given the upregulation of oncogenic kinases and downregulation of tumor suppressive kinases in cancer, Pin1 overactivation through differential phosphorylation can contribute to oncogenesis. An example of this is PLK1-mediated Pin1 phosphorylation on Ser65. This phosphorylation event promotes Pin1 stability by preventing its ubiquitination and subsequent degradation (Pu et al., 2020). PLK1 is a promoter of the G2/M phase transition; overexpression is a negative prognostic marker in various tumors (Liu et al., 2016). Thus, Pin1-stabilization might be one of the many molecular mechanisms by which PLK1 mediates tumorigenesis. Another Pin1 phosphosite that can be altered in cancer is Ser138. Akin to Ser65 phosphorylation, Ser138 phosphorylation by mixed lineage kinase 3 (MLK3) promotes Pin1 activation. MLK3-dependent Pin1 phosphorylation has been shown to promote Pin1 nuclear translocation and activation of oncogenic GLI1 in pancreatic cancer cells (Viswakarma et al., 2021). In contrast to Ser65 and Ser138, Ser71 phosphorylation by DAPK1 inhibits Pin1 PPIase activity and, subsequently, cellular proliferation in triple-negative breast cancer (TNBC) cells (Lee et al., 2011b). Importantly, DAPK1 is frequently lost and is correlated with increased aggressiveness in several tumor types, including breast and colorectal cancers (Asiaf et al., 2019; Steinmann et al., 2019). With DAPK1 loss, decreased Pin1 inhibition and enhanced tumorigenesis would likely follow.

SUMOylation has been closely associated with cellular proliferation and oncogenesis (Han et al., 2018). Molecularly, the addition of a SUMO group to lysine residues can promote or inhibit protein–protein interactions in addition to regulating protein conformation/function (Wilkinson and Henley, 2010). In the case of Pin1, it is SUMOylated on Lys6 in the WW domain and Lys63 in the PPIase domain by SUMO1. Such modifications are inhibitory to Pin1 function since they result in the occlusion of both sites that Pin1 requires to bind to its phosphorylated substrates. These inhibitory modifications can be reversed by SUMO protease 1 (SENP1), which binds to Pin1 and promotes de-SUMOylation. Accordingly, SENP1 has been shown to promote Pin1-dependent oncogenesis, and SENP1 and Pin1 expression levels are positively correlated in breast cancer tissue (Chen et al., 2013; Chen et al., 2020).

### Pin1 mutations in cancer

Mutations in both the regulatory and protein-coding regions of Pin1 can affect its levels and function. Carriers of the −842G>C SNP in the Pin1 promoter have been associated with decreased Pin1 expression levels and a decreased risk for cancer (Li Q. et al., 2013). Another promoter SNP (−667T>C) has been associated with a lower risk for developing nasopharyngeal carcinoma but an increased risk for oral squamous cell carcinoma and hepatocellular carcinoma (Yao et al., 2014; Huang et al., 2016). Data obtained from the COSMIC database by El Boustani et al. (2019) from over 40,000 cancer patients showed that 32 somatic Pin1 mutations occurred in 29 residues; 25 mutations were in the PPIase domain, with 5 and 2 mutations in the WW domain and flexible linker, respectively. Intriguingly, 17 of the mutations were predicted to be pathogenic and promote tumorigenesis, whereas only 4 were predicted to be deleterious (El Boustani et al., 2019). However, the detailed functional consequences of such mutations are unknown and warrant further investigation; it would be interesting to uncover the molecular mechanisms by which these mutations mediate Pin1-dependent tumorigenesis.

In contrast to its multifaceted roles as a tumor-promoting enzyme, Pin1 has also been described as a “conditional” tumor suppressor. The loss of Pin1 in mouse endothelial fibroblasts has been shown to destabilize Myc and cyclin E, which are degraded by the tumor suppressor FBW7, and promote genomic instability in a p53-dependent manner *in vitro* (Yeh et al., 2004; Yeh et al., 2006; Yeh and Means, 2007). This led to the hypothesis that Pin1 may also act as a tumor suppressor. However, the same group later reported that Pin1 stabilizes Myc and enhances its DNA-binding propensity in cancer cells (Farrell et al., 2013). Although these controversial results might suggest that Pin1 can have context-dependent tumor suppressive functions, there is far more evidence to suggest that Pin1 plays a pro-tumor role in various tumors. Particularly, Pin1 knockout mice are highly resistant to tumorigenesis, even with accompanying oncogenic alterations in MYC, HER2, RAS, and p53 (El Boustani et al., 2019).

Overall, by regulating numerous pathways that are central to transmitting oncogenic signals, Pin1 plays a fundamental role in promoting tumorigenesis. This role of Pin1 in the regulation of numerous cancer signaling pathways positions Pin1 as a “master cancer regulator.” Perhaps more importantly, Pin1 is also a master regulator of cancer stem cells (CSCs), and it is here that Pin1 plays functional roles that contribute to cancer aggressiveness and therapeutic resistance.

### Pin1 in EMT and cancer stem cells

Cancer stem cells (CSCs) are regarded as tumor-initiating cells with prominent invasive, metastatic, and drug-resistant phenotypes (Yu et al., 2012). Like normal stem cells, CSCs have the capacity to self-renew and differentiate into various cell types, thereby promoting phenotypic heterogeneity (Atashzar et al., 2020). Although tumor heterogeneity limits the chance of therapeutic success—simply by creating a diverse cell population that might differ in treatment response—CSCs are also inherently drug-resistant. This can be attributed to their ability to remain dormant, increased capacity for DNA repair, and higher expression of drug efflux transporters (Steinbichler et al., 2018).

Accumulating evidence now suggests that the function of CSCs is closely related to the epithelial-to-mesenchymal transition (EMT) (Tanabe et al., 2020). The EMT is a reversible process where epithelial cells are epigenetically reprogrammed to gain the phenotype of mesenchymal cells, which share morphological features with fibroblasts and display enhanced migratory and invasive characteristics (Thiery et al., 2009; Celià-Terrassa and Jolly, 2020). In addition to its relationship with cancer stemness, the EMT has been shown to have a significant impact on the tumor microenvironment (TME) and is closely associated with drug resistance and enhanced metastatic capabilities (Erin et al., 2020). Thus, like CSCs, cancer cells displaying an EMT phenotype are correlated with increased aggressiveness and worse clinical outcomes.

A central reason for tumor recurrence following treatment can be attributed to the persistence of mesenchymal-like cancer stem cells (Houthuijzen et al., 2012). Many therapeutic interventions can successfully eradicate differentiated tumor cells; however, a big problem with conventional targeted/chemotherapeutic approaches is that they fail to target CSCs (Arima et al., 2020; Yang et al., 2020). As a result, the CSC population can remain and repopulate the tumor following treatment cessation (Yan and Liu, 2016). To achieve tumor eradication and prevent metastatic dissemination, it is essential to elucidate druggable targets that promote EMT and CSCs.

**FIGURE 2:**
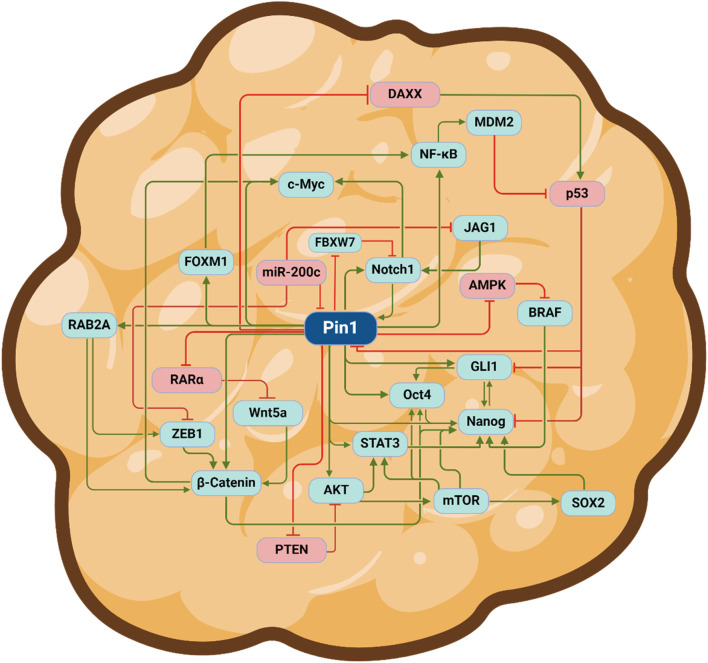
Pin1 regulates various oncoproteins and tumor suppressor proteins to sustain a cancer stem cell phenotype. Depiction of a cancer stem cell. Green arrows indicate positive regulation (activation), and red lines represent negative regulation (inhibition). Abbreviations: DAXX, death domain-associated protein; MDM2, mouse double minute homolog; NF-ĸB, nuclear factor kappa-light-chain-enhancer of activated B cells; GLI1 glioma-associated oncogene homolog 1; JAG1, jagged1; AMPK, AMP-activated protein kinase; BRAF, v-raf murine sarcoma viral homolog B1; SOX2, SRY-box 2; mTOR, mammalian target of rapamycin; STAT3, signal transducer and activator of transcription 3; Oct-4, octamer-binding transcription factor 4; PKB, protein kinase B; PTEN, phosphatase and tensin homolog; ZEB1, zinc finger E-box binding homeobox 1, Wnt5a, wingless/integrated-5a; RARα, retinoic acid receptor α; FOXM1, forkhead box protein M1; c-myc, c-myelocytomatosis oncogene; RAB2A, member Ras oncogene family; FBXW7, F-box and WD repeat domain containing 7; Notch1, neurogenic locus notch homolog protein 1. The figure was generated using BioRender^®^.

Many of the 70 oncoproteins and 30 tumor suppressors that Pin1 regulates are involved in cell stemness; when dysregulated, they play a prominent role in the sustenance of CSCs (Zhou and Lu, 2016). First, Pin1 directly interacts with and stabilizes nanog—a transcription factor crucial for the self-renewal of embryonic stem cells (ESCs) (Moretto-Zita et al., 2010). The disruption of the Pin1–nanog interaction inhibits the ability of ESCs to undergo self-renewal and form teratomas in nude mice (Moretto-Zita et al., 2010). Pin1 also directly interacts with and regulates the stability, nuclear localization, and transcriptional capabilities of c-myelocytomatosis (c-MYC)—a transcription factor implicated in many cancers that prominently contributes to cell proliferation, survival, and stemness (Annibali et al., 2014). Increased c-MYC activity is associated with increased aggressiveness, blocked neuronal differentiation, and enhanced self-renewal propensity of GBM-tumor-initiating cells (Zheng et al., 2008). Ablating Pin1 enzymatic activity with Sulfopin, a selective Pin1 inhibitor, was successful at blocking MYC-driven tumors like pancreatic ductal adenocarcinoma (PDAC) and triple-negative breast cancer, *in vitro* and *in vivo* (Dubiella et al., 2021). Pin1 also interacts with and isomerizes the phosphorylated Ser^12^-Pro motif of octamer-binding transcription factor 4 (Oct4)—another ESC regulatory TF—leading to increased Oct4 stability and transcriptional activity (Nishi et al., 2011). Importantly, Oct4 is highly expressed in CSCs from numerous cancers, including breast, prostate, and hepatocellular carcinoma (Zeineddine et al., 2014; Wu et al., 2015). Although increased Oct4 expression is associated with enhanced resistance to chemo-, radio-, and targeted therapies, Oct4 knockdown sensitizes glioma cells to treatment with temozolomide chemotherapy (Ikushima et al., 2011; Mohiuddin et al., 2019). As mentioned previously, Pin1 also interacts with phosphorylated Notch1 and enhances ϒ-secretase-dependent Notch1 cleavage/activation. Notably, Notch1 expression is increased in CSCs—relative to non-stem tumor cells—from cancers such as renal cell carcinoma, TNBC, and glioma (Zhang et al., 2008; Xiao et al., 2017; Sui et al., 2020). However, the inhibition of Notch1 signaling with the Notch1/2 small-molecule inhibitor MRK-003 or its endogenous inhibitor Numb decreased RCC stem-like characteristics such as self-renewal, chemoresistance, tumorigenesis, and migratory potential (Xiao et al., 2017). Pin1 signaling in cancer stem cells is a highly intricate and interconnected process. Pin1 activity results in the activation and inactivation of pathways responsible for promoting and inhibiting a stem-like state, respectively. Moreover, numerous Pin1 substrates interact with each other to further enhance a stem-like phenotype (Figure 2).

Numerous studies have also shown that Pin1 plays a prominent role in promoting the EMT. Molecularly, Pin1 directly interacts with β-catenin—a well-known promoter of EMT—blocks its interaction with adenomatous polyposis coli protein (APC), and enhances β-catenin stability. Therefore, Pin1 inhibition results in robust β-catenin degradation (Ryo et al., 2001; He, 2006). Indirectly, Pin1 overexpression results in enhanced β-catenin nuclear accumulation, which leads to the transcription of EMT-related genes such as fibronectin (Chen and Igumenova, 2023). Pin1 is also responsible for activating signal transducer and activator of transcription 3 (STAT3) and p65/NF-ĸB—transcription factors known for their role in promoting EMT (Nakada et al., 2019). Importantly, Pin1-depleted cells displayed significantly decreased STAT3 and NF-ĸB activation, which led to decreased vimentin, snail, and zeb-2 expression (Nakada et al., 2019). Coincidentally, high Pin1 expression is associated with increased invasion/metastasis and is an independent prognostic factor for poor clinical outcomes in gall bladder cancer (GBC) (Nakada et al., 2019).

Astonishingly, Pin1 expression is elevated approximately 6-fold higher in CD24^−^/CD44^+^ breast cancer cells (CSCs) relative to non-CD24^−^/CD44^+^ breast cancer cells (non-stem cancer cells) (Luo et al., 2014). Phenotypically, Pin1 overexpression transforms regular mammary epithelial cells into stem-like tumor cells with enhanced tumorigenicity, whereas Pin1 inhibition or knockdown significantly impairs breast CSC expansion, mesenchymal marker expression, tumor initiation, self-renewal potential, and metastasis (Ryo et al., 2001; Zhang et al., 2008; Kim et al., 2009; Ikushima et al., 2011; Tong and Jiang, 2015; Xiao et al., 2017; Chen et al., 2019; Celià-Terrassa and Jolly, 2020; Sui et al., 2020). Given that Pin1 plays an essential role in the maintenance of a cancer stem-cell phenotype, combination therapies that include a Pin1 inhibitor are an attractive approach to sensitizing drug-resistant CSCs to treatment with cytotoxic agents that were not effective in the absence of Pin1 inhibition.

### Pin1 and the immunosuppressive tumor microenvironment

For a tumor to successfully grow, it needs to be undetectable by the host immune system or grow at a rate that exceeds the rate of immune clearance (Kim and Cho, 2022). For normal cells, the presentation of intracellular peptides on major histocompatibility complex 1 (MHC I) allows the immune system to differentiate self-antigens from those that are foreign (Antoniou and Powis, 2008). At the initial stages of transformation—prior to widespread mutational events—neoplastic or pre-neoplastic cells are often indistinguishable from physiologically normal cells and can thus remain unhindered by the immune system (Wu et al., 2020). However, upon tumoral progression, genomic instability and the resulting abnormal protein expression often follow (Prost et al., 2021). Although rapid mutational events can be beneficial for a tumor by promoting alterations that confer a selective growth advantage, they can be detrimental from the perspective of immunosurveillance. If peptides presented on MHC I are altered enough from self, they can be recognized as foreign, and a cytotoxic immune response can be activated to eliminate the tumor (Snyder and Chan, 2015). Thus, with an increased mutational burden, one might expect an increase in the immune response. However, cancer cells often develop mechanisms to evade immune recognition (Schreiber et al., 2011).

Tumor immunosuppression is thought to result from a decrease in immunocyte infiltration and/or function and increased infiltration of immunosuppressive cells in the TME (Yang et al., 2019). Immuno-evasive mechanisms in tumors include MHC downregulation, the production of immunosuppressive cytokines, blocking immune cell infiltration by fibroblast proliferation, and perhaps the most famous of all: immune checkpoint activations (He and Xu, 2020; Sahai et al., 2020; Taylor and Balko, 2022; Tie et al., 2022). Increased tumoral immune response is significantly correlated with longer overall survival in several cancer types, including but not limited to breast, cervical squamous cell carcinoma, sarcoma, and melanoma (Liu, 2019). As a result, immunotherapy—which aims to reactivate the immune system to promote tumor destruction—has been deemed a breakthrough in the world of oncology (Wang et al., 2022). However, the efficacy of immunotherapy is limited by resistance mechanisms (Liu et al., 2019). Thus, it is essential to elucidate therapeutic approaches that might sensitize tumors to immunotherapies.

Pin1 plays a well-established role in the activation of the host immune response by promoting type I interferon-mediated immunity and activating and promoting the nuclear import of p65/RelA/NF- κB (Ryo et al., 2003; Tun-Kyi et al., 2011). However, recent studies suggest that Pin1 plays a prominent role in cancer-related immunosuppression (Koikawa et al., 2021).

In PDAC, tumor heterogeneity and a desmoplastic and immunosuppressive TME are large contributors to therapeutic resistance and the near-universal mortality rate seen in these PDAC patients (Masugi, 2022). The dense, fibrotic microenvironment can mainly be attributed to the overactivation of cancer-associated fibroblasts (CAFs) (Antoniou and Powis, 2008). In the TME, CAFs secrete ECM proteins and promote crosslinking of fibrillar collagen matrices that essentially “encapsulate” the tumor, promoting hypoxia and limiting molecular exchange and immunocyte infiltration (Zeltz et al., 2020). Importantly, in PDAC, Pin1 overexpression in CAFs is correlated with a desmoplastic and immunosuppressive TME and worse clinical outcomes. Pin1 inhibition in patient-derived orthotopic xenograft mice limits the propensity of CAFs to promote a desmoplastic and immunosuppressive microenvironment (Koikawa et al., 2021). Future investigations aim to elucidate the mechanisms by which Pin1 mediates CAF-dependent desmoplasia.

Programmed death-ligand I (PD-L1) expression is an immune checkpoint transmembrane protein involved in immunoregulation. Normally, it is expressed on antigen-presenting cells to regulate ongoing inflammation and auto-reactive T cells (Ghosh et al., 2021). However, cancer cells aberrantly express PD-L1 to evade immune recognition. Interestingly, Pin1 promotes the lysosomal-mediated degradation of PD-L1; thus, Pin1 inhibition can increase PD-L1 expression (Koikawa et al., 2021). As a result, Pin1 inhibitors synergize with anti-PD-1 therapy in PDAC and colorectal cancer (Koikawa et al., 2021; Li et al., 2024). Recent studies suggest that patients with elevated PD-L1 respond better to PD-1 blockade (Herbst et al., 2014). Moreover, elevating PD-L1 expression using various experimental approaches has been shown to enhance the ICI response (Zhang et al., 2018). However, the mechanism by which increased PD-L1 expression leads to enhanced anti-tumor immunity warrants further investigation.

Pin1 inhibition in PDAC decreased collagen deposition and “cleared” the desmoplastic TME, thereby promoting molecular exchange, increased CD8^+^ T-cell infiltration, and increased PD-L1 expression on tumor cells. Resultantly, PDAC mice were sensitized to treatment with αPD-L1 immunotherapy in combination with gemcitabine chemotherapy. Astonishingly, the combination of Pin1 inhibitors and immunochemotherapy achieved over 85% tumor eradication after 1 year even with only 4 months of the treatment, compared with 0% in the immunochemotherapy group alone (Koikawa et al., 2021).

### Overview of key small-molecule Pin1 inhibitors

Most gene targets for cancer therapies are pan-essential genes, and these therapeutics are often limited due to dose-dependent toxicity. The dose of these therapeutics needs to be finely attenuated to mitigate side effects and have a high clinical failure rate with some of the similar pitfalls of chemotherapy (Chang et al., 2021). The non-essentiality of Pin1 offers further potential for combination therapies as it could reduce the effective dose, limit on-target toxicity, and broaden cytotoxic effects observed with chemotherapy. Drug-resistant cancers are becoming more prevalent, and they continue to be the biggest clinical challenge for cancer treatment and a principal roadblock in cancer drug development. The mechanisms through which cancers acquire resistance are multifaceted, but Pin1 is uniquely situated as a master regulator at the center of a complex network of signaling pathways that drive cancer drug resistance. Small-molecule inhibitors have been used to probe the effect of Pin1 inhibition on resistance, and in many cancer types, it has either reversed resistance or sensitized the cancer for treatment with other therapeutic agents. A detailed summary of the use of juglone, EGCG, ATRA, and ATO with other therapeutic agents and the effect of the combination therapy on cancer drug resistance in different cancer types is summarized in Wu et al. (2022). The shallow nature of the Pin1 active site and the phosphate-binding pocket, which prefers negatively charged groups, has made it challenging to design cell-permeable inhibitors that bind with sufficient potency. Over the past two decades, different strategies have been used to target Pin1 with varying degrees of success, but the scope of this review is focused on small-molecule inhibitors of Pin1. The molecular structures and other information for selected inhibitors, including those discussed in this review, are presented in Table 1.

**TABLE 1:** Selected cell active Pin1 inhibitors and their properties.

Pin1 inhibitor	Chemical structure	Identification method	IC_50_ (µM)	Pin1 inhibitory mechanism and specificity	System in which Pin1 inhibition was observed	Reference
Juglone	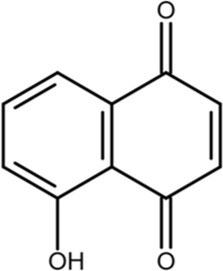	Low-throughput enzymatic assay	Not determined	Covalently modifies the active site Cys in Pin1, parvulins, and other proteins	*In vitro*	Holford (2018)
PiB	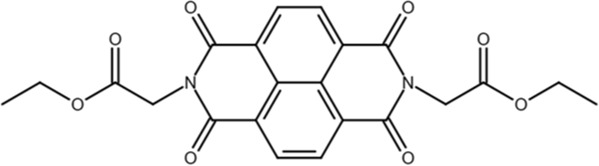	Low-throughput enzymatic assay	1.5	Inhibits Pin1, parvulin, and likely other proteins	*In vitro* and cell models	Zheng (2017)
DTM	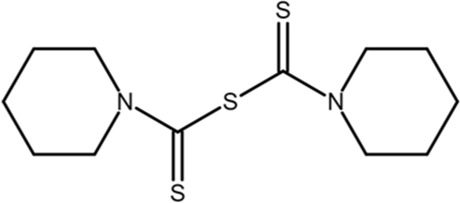	Low-throughput enzymatic assay	4.0	Inhibits Pin1 and likely others	*In vitro* and cell models	Tatara et al. (2009)
Phenyl-imidazoles	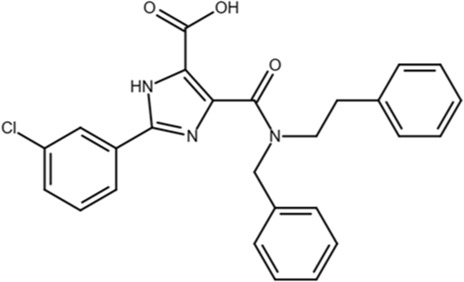	Structure-based design	0.8	Binds to the Pin1 active site, confirmed by crystal structure studies	*In vitro* and cell models	Chao et al. (2001), Potter et al. (2010a)
EGCG	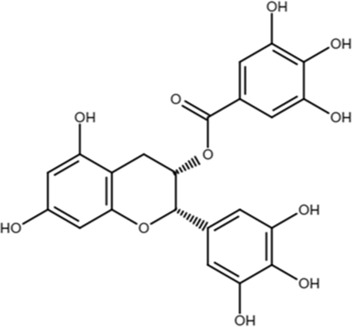	Phenotypic association	22.0	Binds to the WW domain and PPIase domain of Pin1, confirmed by crystal structure studies; also binds to many other targets	*In vitro*, cell models, and mouse models	Urusova et al. (2013)
Buparvaquone	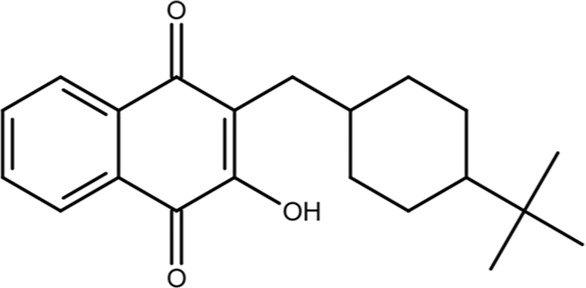	Structural similarity	Not determined	Covalently modifies the active site Cys in TaPin1 secreted by intracellular parasites, with a drug-resistant TaPin1 mutant identified	*In vitro*, cell models, and zebrafish	Marsolier et al. (2015)
ATRA	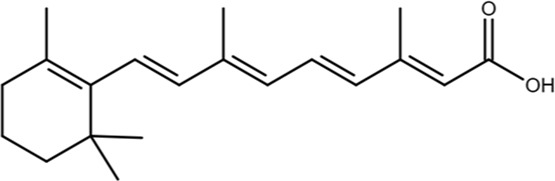	Mechanism-based high-throughput screen	0.8	Binds to the active site and induces the degradation of active Pin1 selectively in cancer cells by mimicking a substrate, confirmed by crystal structure studies; also binds to RAR/RXR	*In vitro*, cell models, mouse models, and human patients	Wei et al. (2015)
Compound 17	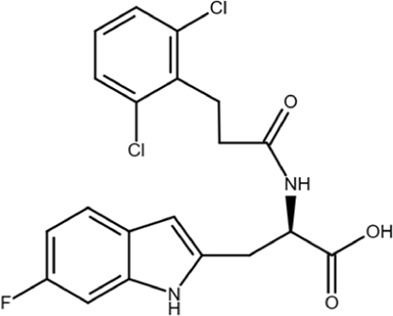	Structure-guided drug development	Not determined	Non-covalent inhibitor that forms H-bonds with key active cite residues	*In vitro*	Guo et al. (2014), Russo Spena et al. (2018)
ATO	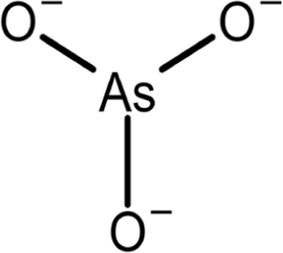	Functional connection	0.1	Binds to the Pin1 active site in the Pro-binding pocket and induces Pin1 degradation in synergy with ATRA, confirmed by crystal structure studies; also binds to other proteins	*In vitro*, cell models, and mouse models	Kozono et al. (2018)
KPT-6566	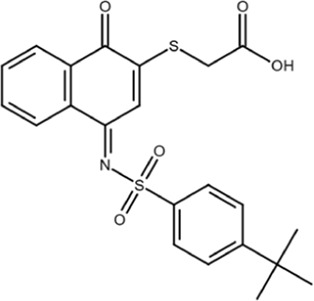	Structure-based virtual screen	0.6	Covalently modifies the active site Cys in Pin1 and induces Pin1 degradation; also induces oxidative stress	*In vitro*, cell models, and mouse models	Campaner et al. (2017)
API-1	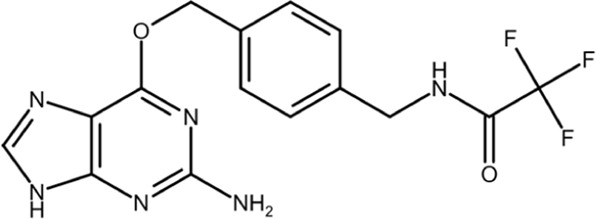	Structure-based virtual screen	72	Binds to the Pin1 active site by interacting with K63, R69, C113, M130, Q131, and H157 based on molecular modeling and site-directed mutagenesis	*In vitro*, cell models, and mouse models	Pu et al. (2018)
BJP-06–005–3	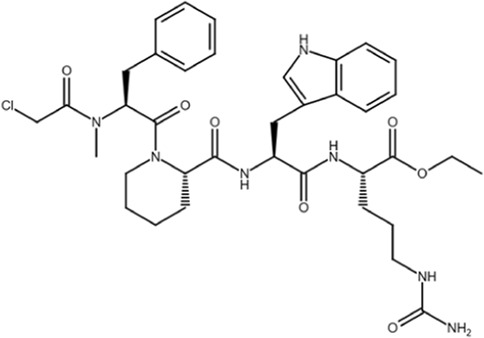	Structure-based design	0.007	Covalently modifies the active site Cys in Pin1 derived from 1 nM peptide inhibitor pTide, confirmed by crystal structure studies, and induces Pin1 degradation	*In vitro* and cell models	Wildemann et al. (2006), Zhang et al. (2007), Pinch et al. (2020)
Sulfopin	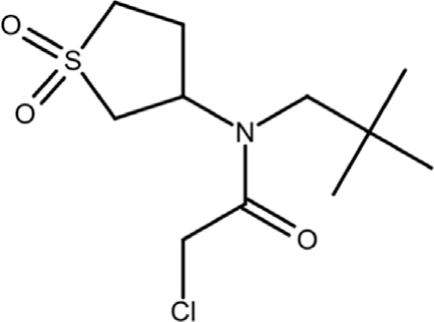	Low-throughput binding screen	0.038	Covalently modifies the active site Cys and targets the ATO-binding pocket in Pin1, confirmed by structure; induces Pin1 degradation; no other known targets; no notable toxicity	*In vitro*, cell models, human organoids, and mouse models	Dubiella et al. (2021), Koikawa et al. (2021)
AG17724 encapsulated using DNA-barcoded micellular system	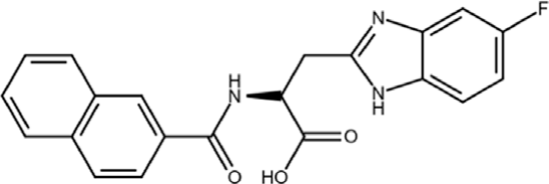	Structure-based design selectively delivered to CAFs		Binds to the active site and induces the degradation of active Pin1 selectively in cancer-associated fibroblasts	Cell models, human organoids, and mouse models	Guo et al. (2014), Liu et al. (2022)

Juglone was the first inhibitor discovered in 1998 from a collection of metabolites tested for inhibitory effects on parvulin. Juglone was shown to bind human Pin1 through covalent modification at cysteine 113, with the unsaturated carbonyl system reacting with the sulfhydryl group of the cysteine residue (Hennig et al., 1998). This reaction at the key catalytic cysteine leads to an irreversible loss in Pin1 enzyme activity with treatment at high doses, decreasing Pin1 protein levels and suppressing cancer cell proliferation in breast, lung, and prostate cancers (Zeltz et al., 2020; Ghosh et al., 2021). Juglone also plays a role in the regulation of other proteins as it inhibits RNA polymerase II transcription by rapidly blocking the formation of preinitiation complexes (Chao et al., 2001). Despite the *in-vivo* validation of the anti-cancer effects of juglone, its lack of specificity to Pin1 and the nature and diversity of its numerous off-target interactors limit the use of juglone as a potential cancer therapeutic.

PiB was identified in 2003 by screening 1,000 chemically synthesized compounds for Pin1 inhibition using a peptide in conjunction with a Pin1-PPIase activity assay, which produced some lead molecules. A round of synthesis and selection based on the initial hits produced a series of compounds with PiB as the most potent competitive inhibitor with an IC_50_ of 1.2 μM in HCT116 cells and less than 10 μM in a range of other cancer cell lines (Uchida et al., 2003). PiB competes with the substrate for binding to the active site of Pin1. Treatment with either juglone or PiB showed that Pin1-inhibition results in reduced Nanog expression through reduced transcription and increased proteasomal degradation. With respect to Nanog degradation, active Pin1 stabilizes Nanog through a ubiquitin-dependent mechanism (Moretto-Zita et al., 2010), and inhibition of Pin1 promotes the degradation of Nanog. In mouse embryonic stem cells (ESCs), Pin1 inhibition reduced tumor size by decreasing the self-renewal and teratoma-forming potential of ESCs in immunodeficient hosts (Moretto-Zita et al., 2010).

TME-001 was identified as a potent Pin1 inhibitor in 2011 through a real-time florescence-based high-throughput screen and competitively inhibited Pin1 by binding to the PPIase domain. In HeLa cells, TME-001 treatment suppressed cell proliferation and exhibited an IC_50_ of 6.1 μM, with an effective dose having no cytotoxic effects (Mori et al., 2011). Purportedly, the imidazole nitrogen atoms of TME-001 form hydrogen bonds with Cys113 and Ser 154, with the phenyl ring occupying the hydrophobic pocket near the Pin1 active site (Mori et al., 2011).

Given the strong pharmaceutical interest in the potential of a Pin1 inhibitor as a novel drug, Pfizer and Vernalis invested in their own Pin1 inhibitor research programs in 2009 and developed some inhibitors using a structure-based drug design approach to target the phosphate-binding pocket of Pin1. The Pfizer Pin1 program started by screening more than one million compounds through both a fluorescence polarization-based binding assay and a Pin1 enzymatic assay, but it did not produce any viable hits (Guo et al., 2009; Dong et al., 2010). Instead, they used a Pin1 crystal structure and knowledge of other PPIase inhibitors to develop compounds, some with sub-micromolar inhibitory activity, placing them among the highest affinity inhibitors (Dong et al., 2010; Guo et al., 2014). Despite the significant investment and effort put into the development and optimization of these inhibitors, most molecules were poorly active or even inactive in cells due to the poor cell permeability inherent in lead molecules that contained a polar phosphate or carboxylate. On this basis, further clinical development was not pursued. Vernalis learned from Pfizer that high-throughput screening was not the most effective method of finding leads to investigate and instead developed an NMR-based fragment screening platform called SeeDs that identified compounds that competed with ligands for target binding (Potter AJ. et al., 2010). Through an iterative process of identifying hits, designing and testing compounds, and obtaining crystal structures and further design, they found a unique fragment hit that was developed into compounds with nano-molar potency against Pin1, two of which exhibited low micro-molar inhibition in PC3 cells (Potter AJ. et al., 2010). Once again, compounds like the benzimidazole series of inhibitors exhibited promising *in vitro* activity, but poor cell permeability resulted in failed target engagement *in vivo*. Attempts to increase permeability by reducing the polar surface of the molecule resulted in orders of magnitude loss in potency (Potter AJ. et al., 2010). Vernalis tried to restart by screening 900 fragments using an enzymatic assay and ultimately developed a series of inhibitors based on phenyl-imidazoles that exhibited sub-micro-molar IC_50_ and suppressed prostate cancer cell growth (Potter AJ. et al., 2010).

All-*trans* retinoic acid (ATRA) was identified as a non-covalent Pin1 inhibitor in 2015 through a mechanism-based high-throughput screen of a library of 8,200 compounds (Wei et al., 2015). A fluorescence polarization binding assay was used with a high-affinity fluorescently labeled peptide to look for compounds that competed for binding to active Pin1. Both *cis-RA* and *trans-RA* bind to Pin1, but all *trans*-RA was about twice as potent as the *cis* isomer *in vitro*. Photoaffinity labeling showed ATRA bound directly to Pin1 with a K_d_ of 0.8 μM, and PPIase activity assays and FP-based competition assays showed that ATRA inhibited activity with a K_i_ of 0.82 μM. Co-crystallization and structure-determination revealed that the carboxylic acid group of ATRA forms salt bridges with residues K63 and R69, which are critical residues for binding phosphate group, and the trimethylcyclohexene aromatic ring slots into the hydrophobic binding the pocket in the Pin1 activity site. Through this binding mechanism, ATRA mimics the natural pSer/Thr-Pro substrates by occupying the proline-binding pocket and forming similar interactions with the phospho-binding site within the Pin1 active site (Wei et al., 2015). Unlike PiB and juglone, ATRA treatment in cells leads to the degradation of Pin1 and exhibits anti-cancer properties against TNBC and acute promyelocytic leukemia (APL); however, in TNBC mouse models, ATRA treatment showed only moderate antitumor activity (Wei et al., 2015). In comparison to the previously developed inhibitors, ATRA did not exhibit the same issues with cell permeability and used a novel binding mechanism, which warrants further exploration as new inhibitors designed to mimic the natural substrate could be a promising strategy for novel Pin1-inhibitor development.

Arsenic trioxide (ATO) is a known and approved treatment option for APL, but the mechanism behind its anticancer effects is unknown. Given that Pin1 inhibition and degradation are behind the effects of ATRA on APL, it was hypothesized that ATO could also interact with Pin1 in some manner. Through NMR and crystallographic analysis, it was determined in 2018 that ATO binds to the prolyl-binding pocket of Pin1 and competitively inhibits its PPIase activity with a K_i_ of 0.116 μM; furthermore, inhibition by ATO induces proteasome-dependent Pin1 degradation via a novel non-covalent mechanism (Kozono et al., 2018). Not only can ATRA and ATO act independently to inhibit and degrade Pin1, but in combination they work synergistically for enhanced anti-cancer effects. ATRA increases the cellular uptake of ATO by a dose-dependent increase in the expression of AQP9, a transmembrane protein involved in the arsenic uptake pathway, while also directly inhibiting Pin1 (Kozono et al., 2018). Combination treatment of ATRA and ATO mostly cures APL, and in TNBC mice, this drug combination resulted in better inhibition of tumor growth than treatment using either compound alone. Furthermore, ATRA not only increases the cellular availability of ATO but also reduces its overall toxicity, allowing for higher-dose treatment.

KPT-6566 is a covalent Pin1 inhibitor that modifies C113 with the addition of a sulfanyl-acetate group via a disulfide bond; this reaction releases a quinone-mimicking drug that generates reactive oxygen species (ROS) and damages DNA, further contributing to Pin1-mediated cell death in cancer cells (Campaner et al., 2017). A mechanism-based screen of a 200,000-compound library was filtered down to nine candidates based on docking scores, drug-likeness, and pharmacological properties, with the goal of finding a compound that binds covalently at C113. When tested in cells, KPT-6566 was the most potent, with an IC_50_ of 0.64 μM. It is an aromatic compound containing a polycyclic hydrocarbon with a sulfanyl-acetic acid and a tert-butylphenylsulfonamide moiety (Campaner et al., 2017). KPT-6566 is a Pin1-specific covalent inhibitor that does not bind to other PPIases (specifically FKBP4 and PPIA), and treatment with low micromolar concentrations inhibited colony formation in MDA-MB-231 cells. Furthermore, KPT-6566 curbed the self-renewal of breast cancer stem cells (CSCs) with treatment inhibiting mammosphere formation and decreasing the levels of three different stem cell markers. KPT-6566 promotes the proteasomal degradation of Pin1, and its binding releases a by-product that triggers oxidative stress responses, resulting in an increase in endogenous ROS levels in MDA-MB-231 and PC3 cells and inducing DNA damage in a Pin1-dependent manner (Campaner et al., 2017). KPT-6566 treatment suppresses cancer cell invasion and CSC maintenance and causes both a decrease in cancer cell proliferation and an increase in the number of dead cells, indicating that both Pin1-inhibition and the acute increase in ROS and induction of DNA damage together contribute to the observed effect on cancer cell viability. In mice injected with MDA-MB-231 cells, treatment with KPT-6566 significantly reduced metastatic growth with no signs of organ toxicity (Campaner et al., 2017). Normal cells have lower Pin1 expression than cancer cells; therefore, the Pin1-dependent cytotoxic effects of KPT-6566 could be used to specifically target cancer cells.

6,7,4′-Trihydroxyisoflavone (6,7,4′-THIF) is a metabolite that inhibits esophageal cancer growth and directly binds and inhibits Pin1 PPIase activity (Lim et al., 2017). Docking analysis indicated that 6,7,4′-THIF forms hydrogen bonds with both the WW and PPIase domains, and a direct interaction was confirmed *in vitro* in mouse embryonic fibroblasts (MEFs) through immunoprecipitation. 6,7,4′-THIF suppressed tumorigenesis in esophageal cancer cell models and induced cancer cell death (Lim et al., 2017). Finally, tumor growth in MEF xenograft mice was suppressed by 6,7,4′-THIF treatment, with a significant decrease in tumor volume.

Sulfopin is a highly selective and potent covalent Pin1 inhibitor that was developed using a novel fragment-based screening and optimization approach, starting with a library of electrophile-containing drug fragments (Dubiella et al., 2021). Given the phosphate-binding pocket of Pin1, previously developed inhibitors would have to use negatively charged moieties to interact with that region, which would limit cell permeability, but Sulfopin instead exploits direct interactions with the pocket (Dubiella et al., 2021). Sulfopin binds covalently to C113 with a K_i_ of 17 nM and 211 nM, as determined through fluorescence-polarization assays and PPIase enzymatic activity-based assays, respectively. From a co-crystal structure, it was shown that the sulfolane ring occupies the hydrophobic substrate-binding pocket, while the sulfolane oxygens form hydrogen bonds with the backbone amide of E131 and the imidazole NH of H157, mimicking the similar bonding pattern of ATO (Dubiella et al., 2021). Sulfopin is highly selective for Pin1, inhibits Pin1 in multiple cell lines, including MDA-MB-231 and HCT116, reduces tumor progression and tumor volume, and confers a significant survival benefit in a dose-dependent manner in a murine model of neuroblastoma. However, the cell viability effects of Sulfopin in cancer cell lines were modest, but the dose-limiting toxicity was not reached, so future research with prolonged treatments at higher doses or in conjunction with other treatment options could work synergistically for more pronounced suppression of cancer cell growth. Pancreatic ductal adenocarcinoma (PDAC) is a very aggressive cancer that is infamously resistant to available treatment options, including chemotherapy, different targeted therapies, and immunotherapy; the resistance to treatment of PDAC is in part due to its unique immunosuppressive TME (Koikawa et al., 2021). PDAC was shown to be eradicable for the first time by targeting Pin1, which renders the immune “cold” tumor environment “hot” and sensitizes PDAC to immunochemotherapy. In this approach, Sulfopin, or a combination of ATRA and ATO were used to inhibit Pin1, and it highlights the potential of potent Pin1-inhibitors for clinical treatment either on their own or in combination with immunochemotherapy to render aggressive and resistant cancers curable (Koikawa et al., 2021). In summary, Sulfopin is a promising inhibitor that works both *in vitro* and *in vivo* to inhibit Pin1, and its activity was validated in different physiologically relevant disease models where it conferred significant anti-cancer effects.

## Conclusion

Cellular signaling is a complicated network that regulates many different proteins and protein products. It is dynamic and constantly responds to changes in external stimuli and internal factors such as metabolism, cell programming, stress, or genomic instability. A key component of this network is protein phosphorylation, and many oncogenic pathways use proline-directed phosphorylation to drive tumorigenesis. Pin1 is a master regulator of the structure and functions of many phosphorylated proteins; it is overexpressed in most cancers and regulates numerous cancer-driving pathways by specifically isomerizing a key phospho-motif. Cancer successfully uses Pin1’s role to drive multiple oncogenic pathways through stabilizing oncogenes while turning off tumor suppressors. In cancer stem cells, Pin1 stabilizes key transcription factors, thus promoting cell stemness, which drives the retention of a cancer stem-cell phenotype. Pin1 inhibition has been shown to effectively sensitize tumors in immunosuppressive environments, making them amenable to treatment. Therefore, targeting Pin1 activity presents an opportunity to attack cancer development through multiple downstream oncogenic targets simultaneously, curb the expansion of CSCs, and overcome immunosuppressive tumor environments. This review outlined some key Pin1 small-molecule inhibitors and some of their validation through different cancer models, but they all have their limitations. There is still a strong demand for the development of highly potent, Pin1-specific small-molecule inhibitors that are cell-permeable and effectively degrade Pin1. Pin1 inhibitors have a great potential to be used not only as an anticancer drug on their own but also in combination with existing treatment options like chemotherapy, thus leading to the clinical development of novel anticancer therapeutics.
